# ASSOCIATION BETWEEN VIOLENT CRIME INCIDENT PROXIMITY AND COGNITIVE FUNCTION IN OLDER AFRICAN AMERICANS

**DOI:** 10.1093/geroni/igac059.1924

**Published:** 2022-12-20

**Authors:** Genesis Tan, Alejandro Gimenez-Santana, Zuzanna Osiecka, Darlingtina Esiaka, Bernadette Fausto, Mark Gluck

**Affiliations:** Rutgers University - Newark, Newark, New Jersey, United States; Rutgers University - Newark, Newark, New Jersey, United States; Rutgers University - Newark, Newark, New Jersey, United States; Robert Wood Johnson Medical School, Rutgers University, Newark, New Jersey, United States; Rutgers University - Newark, Newark, New Jersey, United States; Rutgers University - Newark, Newark, New Jersey, United States

## Abstract

Research on Area Deprivation Index (ADI) suggests that the built environment and neighborhood stressors (e.g., violent crime incidents) play a role in later-life cognitive function. However, most of the research linking ADI and cognitive function was conducted on majority White American samples. Further, while ADI is useful in facilitating efficient integration of social determinants of health (SDOH) into models of cognitive aging, it does not account for the impact of micro-level measures of neighborhood stressors on cognitive function. Therefore, the purpose of the current study was to determine whether violent crime incident proximity (VCIP) contributes to later-life cognitive function above and beyond ADI in older African Americans. Participants (N=147; M= 68.34) from an ongoing study, Pathways to Healthy Aging in African Americans—a Rutgers University-Newark community partnership fostered over 16 years of community engagement, health education, and public service—responded to measures of cognitive ability, SDOH, and demographic details. The results show that VCIP is a trending predictor of cognitive performance, when adjusting for age, gender, education, depression, and ADI. The result aligns with our hypothesis that individuals living in areas with greater VCIP will have poorer performance on cognitive tasks. Our findings suggest that for African Americans in an urban setting, hyper-local VCIP appears to be more useful at capturing the impact of neighborhood disadvantage on cognitive decline and Alzheimer’s disease risk. Therefore, for later-life cognitive health in African Americans, it is important to consider micro-level measures of neighborhood stressors such as VCIP.

